# Evaluating the Quality of Competency Assessment in Pharmacy: A Framework for Workplace Learning

**DOI:** 10.3390/pharmacy4010004

**Published:** 2016-01-19

**Authors:** Shailly Shah, Jacqueline E. McLaughlin, Stephen F. Eckel, Jesica Mangun, Emily Hawes

**Affiliations:** 1 Johns Hopkins Bayview Medical Center, 4940 Eastern Ave, Baltimore, MD 21224, USA; sshah82@jhmi.edu; 2 UNC Eshelman School of Pharmacy, University of North Carolina (UNC), Chapel Hill, NC 27599, USA; stephen.eckel@unchealth.unc.edu (S.F.E.); emily_hawes@med.unc.edu (E.H.); 3 UNC Medical Center, University of North Carolina (UNC), 101 Manning Dr, Chapel Hill, NC 27514, USA; jmangun@email.unc.edu

**Keywords:** assessment, evaluation, residents, workplace learning, competency

## Abstract

Demonstration of achieved competencies is critical in the pharmacy workplace. The purpose of this study was to evaluate the quality of the competency assessment program for pharmacy residents at an academic medical center. The competency assessment program (CAP) survey is a validated, 48-item instrument that evaluates the quality of an assessment program based on 12 criteria, each measured by four questions on a scale of 0 to 100. The CAP was completed by residents (*n =* 23) and preceptors (*n =* 28) from the pharmacy residency program between 2010 and 2013. Results were analyzed using descriptive statistics, Cronbach’s alpha, and non-parametric tests. Educational Consequences was the only quality criteria falling below the standard for “good quality.” Participants that completed residency training elsewhere rated the Comparability (0.04) and Meaningfulness (0.01) of the assessment program higher than those that completed residency at the academic medical center. There were no significant differences between resident and preceptor scores. Overall, the quality of the assessment program was rated highly by residents and preceptors. The process described here provides a useful framework for understanding the quality of workplace learning assessments in pharmacy practice.

## 1. Introduction

There is growing emphasis on the demonstration of achieved competencies in the health professions [1]. As health care becomes increasingly complex, pharmacists must ensure that they develop and maintain the clinical knowledge and skills necessary to navigate the health care system and optimize patient outcomes [2]. Competency development in the workplace is an ongoing issue for the profession of pharmacy, with discussions ranging from assessment of core competencies in pharmacy education to competency-based continuing professional development.

Identifying best practices for assessing the achievement of competencies continues to be a priority for pharmacists and pharmacy education. Assessment and learning are intimately related, as what is assessed can influence what is learned [3]. Further, effective assessment can increase learner engagement, motivation, and knowledge. Given the integrated nature of assessment and learning, it is critical that those developing and implementing assessment programs in the workplace understand how employees experience competency assessments and the extent to which the quality of the assessment program encourages or inhibits workplace learning.

The American Society of Health-System Pharmacists (ASHP) defines core competencies for pharmacy residents and endorses a tool that pharmacy faculty can use to document competency achievement [4]. While providing assessment tools and guidelines, ASHP allows each health system to design its own assessment program for pharmacy residents, using a wide range of assessment approaches best suited for the health system (e.g., verbal feedback, midpoint evaluation, portfolio, mentoring). While this approach allows for flexibility and diversity of assessment approaches at residency programs across the country, the quality of the assessment programs in pharmacy resident training remains incompletely understood. Evaluating how residents and preceptors experience an assessment program can highlight gaps between teaching and learning, demonstrate inefficiencies, identify program strengths and weaknesses, and ultimately inform approaches to workplace learning and competency development. Improving assessment programs to accurately measure competency achievement and empower self-regulated learning will enable employers to trust that a pharmacy practice residency certificate holder is competent in defined competencies and would be able to better assess an employee’s strengths and needs.

The purpose of this study was to evaluate the assessment program for pharmacy residents at an academic medical center in the United States. Specifically, this study examined resident and preceptor perceptions of the quality of the assessment program and examined differences in perceptions between groups. This work is an important step towards understanding how the program’s assessment practices align with desired competency outcomes and demonstrates a process for evaluating stakeholder perceptions of assessments in workplace learning.

## 2. Experimental Section

This study took place at a large academic medical center in the United States (greater than 800 beds and approximately 37,500 admissions per year) that trains pharmacy residents in one of 12 different residency programs: a Post-Graduate-Year 1 (PGY1) pharmacy practice residency; 10 Post-Graduate Year 2 (PGY2) pharmacy specialty programs; and a combined PGY1 and PGY2 health system pharmacy administration residency. Pharmacy residency is a competitive training program for graduates holding a Doctor of Pharmacy (PharmD) degree. Since 2007, the residency programs have largely utilized PharmAcademic (formerly known as Resitrak) [5], a McCreeadieGroup web-based tool which enables scheduling and documentation of evaluation of resident learning experiences. Through this platform, residency preceptors are able to utilize a variety of assessment strategies to evaluate and document resident competencies each month, including direct observation of skills and outcomes to documentation of experiences. Residency completion certificates are presented to residents upon verified completion (e.g., marked as achieved) of at least 85% of Resitrak goals by the residency program director.

### 2.1. Data Collection

The Competency Assessment Program (CAP) framework uses 12 quality criteria to evaluate the perceived value of an assessment program: Acceptability; Authenticity; Cognitive Complexity; Comparability; Costs and Efficiency; Educational Consequences; Fairness; Fitness for Purpose; Fitness for Self-Assessment; Meaningfulness; Reproducibility of Decisions; and Transparency (Table 1) [6,7,8,9]. Educational Consequences, for example, concerns the impact of the assessment program on learning and instruction, including how the individuals view the goals of education and adjust their learning activities accordingly. Transparency reflects the extent to which a CAP is clear and understandable, including the purpose, assessors, and scoring criteria while Meaningfulness indicates the value of the CAP and emphasizes the perceived link between the assessment task and personal needs or goals [8]. One strength of this framework is its representation of assessment programs as a comprehensive system, since evaluating one single assessment method fails to fully consider the complex and integrated nature of competency development. To-date, the CAP framework has been implemented and studied in various educational settings, validated in various contexts, and adapted to meet the needs of an institution [6,7,8,9].

**Table 1:** Description of the twelve quality criteria for a Competency Assessment Program (CAP), adapted from Baartman and colleagues [6].

Criterion	Description
*Acceptability*	The extent to which all stakeholders (e.g., students, teachers, employers) approve of the assessment criteria and the way the CAP is carried out
*Authenticity*	The degree of resemblance of a CAP to the future workplace
*Cognitive-Complexity*	The extent to which the assessments reflect the presence of the cognitive skills and enable the judgment of thinking processes
*Comparability*	The extent to which a CAP is conducted consistently and responsibly
*Costs and Efficiency*	The time and resources needed to develop and carry out the CAP, compared to the benefits
*Educational Consequences*	The degree to which the CAP yields positive effects on learning and instruction, and the degree to which negative effects are minimized
*Fairness*	The extent to which learners get a fair chance to demonstrate their competences, for example by limiting assessor bias
*Fitness for Purpose*	The degree to which standards, curriculum, instruction and assessment are aligned
*Fitness for Self-Assessment*	The degree to which a CAP stimulates self-regulated learning of students, including fostering self-assessment and giving and receiving feedback
*Meaningfulness*	The value of the CAP for all stakeholders involved (e.g., students, teachers, employers)
*Reproducibility of Decisions*	The extent to which decisions made from results of CAP are accurate and constant over situations and assessors
*Transparency*	The extent to which the CAP is clear and understandable to all stakeholders (e.g., students, teachers, employers)

The CAP survey is a validated instrument that evaluates these 12 quality criteria, each measured with 4 items on a scale from 0 to 100 [9]. In this study, the CAP survey was adapted to align with the context of the residency assessment program and written for two sets of participants: (a) past and current residents of the residency program (survey #1); and (b) preceptors of the residency program (survey #2). Each survey also included demographic questions (e.g., *role in program, year of residency completion, residency experience outside of the institution,* and *numbers of years of experience*) and a list of assessment methods for respondents to identify as methods currently utilized in the program, chosen based on ASHP’s *Resident’s Guide to the Residency Learning System* (RLS) [5]. The survey went through three rounds of review by the research team for appropriateness and face validity.

In fall 2013, the survey was distributed via email to pharmacy residents in the program between 2010 and 2013 (*n =* 41) and to pharmacy preceptors that precepted residents at any time between 2010 and 2013 (*n =* 53). The timeframe of 2010–2013 was chosen in hopes of having a greater number of participants while limiting recall bias. The survey was left open for a period of 1 month, and two emails were sent as reminders for the survey. Preceptors were also reminded to complete the survey during a staff meeting. All data was collected anonymously.

### 2.2. Data Analysis

Responses with less than 75% completion were excluded from all analyses. Pairwise deletion was used for responses where all 4 indicators for a single quality criterion were left blank and mean substitution was used for other data missing at random. Descriptive statistics were calculated for the components of the residency program, the composite 12 quality criteria, and each individual indicator within each quality criterion. Cronbach’s alpha was calculated for each of the 12 quality criteria, with α > 0.60 considered acceptable and indicating an analysis of the composite quality criteria from each of the four indicator questions to be appropriate [10]. A standard of α ≤ 0.60 was used to indicate that the construct lacked internal consistency. Composite scores for each of the 12 quality criteria were created by averaging the four indicators within the criteria. Due to small sample sizes, comparisons between groups were made using the Mann-Whitney U for 2 groups and Kruskal-Wallis one-way analysis of variance (ANOVA) for 3 or more groups. Group comparisons were made to examine differences in responses between residents and preceptors, between those that completed residency at the institution only and those that completed a residency elsewhere, and between groups based on number of years of precepting, year of residency completion, or experience precepting elsewhere. Descriptive statistics are represented in the text by median, range. Statistical significance was established at α = 0.05. Given the lack of power to detect anything but large differences due to small sample sizes, any p value less than 0.20 was used to generate hypotheses for future research. Survey responses were analyzed with IBM SPSS Statistics for Windows (Version 21.1. Armonk, NY: IBM). The study received exemption from full review by the Institutional Review Board.

## 3. Results and Discussion

### 3.1. Demographic Results

The survey was completed by 23 residents (56.1% response rate) and 28 preceptors (52.8% response rate). Sixteen residents completed PGY1 training at the institution, 20 completed PGY2 training at the institution, and 13 residents completed both PGY1 and PGY2 training at the institution (Table 2). In addition to residency experience at this institution, seven residents completed one year of residency training at a different institution. A majority of respondents participated in residency training at the institution in 2012 (*n =* 14) and 2013 (*n =* 15), with 4 residents receiving training in 2011.

Of the 28 preceptor respondents, nine completed PGY1 and PGY2 training at the institution. Eleven preceptor respondents completed PGY1 and nine completed PGY2 training at the institution, with 14 preceptors completing a residency experience at another institution. In addition to his or her current precepting role, 18 of the 28 preceptors had precepting experience at another institution. Four preceptors reported having less than three years of precepting experience, 13 preceptors between three and 10 years, and 11 preceptors with more than 10 years of precepting experience.

**Table 2:** Demographic characteristics and perceived presence of assessment methods.

Characteristic	Residents (*n =* 23)	Preceptors (*n =* 28)
*Residency Experience*		
PGY1 at the institution	16 (70)	11 (39)
PGY2 at the institution	20 (87)	9 (32)
PGY1 and PGY2 at the institution	13 (57)	9 (32)
Completed a residency elsewhere	7 (30)	14 (50)
Completed residency in 2011	4 (17)	N/A
Completed residency in 2012	14 (61)	N/A
Completed residency in 2013	15 (65)	N/A
Currently practicing at the institution	14 (61)	N/A
*Preceptor Experience*		
Precepted elsewhere	N/A	18 (64)
Precepted less than 3 years	N/A	4 (14)
Precepted 3 to 10 years	N/A	13 (46)
Precepted more than 10 years	N/A	11 (39)
*Assessment Method ^a,b^*		
Verbal Feedback	23 (100)	26 (93)
Assigning Goals and Objectives to Learning Outcomes	19 (83)	23 (82)
Snapshot Evaluations	3 (13)	8 (29)
Mentor Relationship	22 (96)	18 (64)
Quarterly Self Evaluations	18 (78)	13 (46)
Quarterly Program Director Evaluations	16 (70)	11 (39)
Monthly Summative Self Evaluations	22 (96)	20 (71)
Monthly Summative Preceptor Evaluations	21 (91)	21 (75)
Monthly Evaluation of Preceptor	21 (91)	21 (75)
Learning Experience Evaluation	10 (43)	10 (36)
Midpoint Evaluation	16 (70)	23 (82)
Midpoint Self-Evaluation	9 (39)	8 (29)
Observation in Simulated Situations	3 (13)	5 (18)
Observation in Workplace	11 (48)	18 (64)
Portfolio	5 (22)	3 (11)
Proof of Competency Assessments	3 (13)	4 (14)
Assessment Interview	1 (4)	2 (7)

All data presented as *n* (%); ^a^ Assessment Methods drawn from Resident’s Guide to Residency Learning System [5]; ^b^ *Which of the following were included in the residency assessment program during your residency experience.*

When asked to identify the assessment methods utilized within the residency assessment program, all residents agreed that *verbal feedback* was a part of the residency assessment program. At least 90% of residents also agreed that *mentor relationship*, *monthly summative self evaluations*, *monthly summative preceptor evaluations,* and *monthly evaluations of preceptors* was part of the residency assessment program. Although 93% of preceptors agreed that *verbal feedback* was an assessment method used in the program, the remaining methods received less than 90% agreement, with *assigning goals and objectives to learning outcomes* (82%) and *midpoint evaluations* (82%) the only others above 80% agreement. Few residents and preceptors identified *snapshot evaluations, portfolio,* and *assessment interviews* as part of the residency assessment program.

### 3.2. CAP Responses

Table 3 describes the combined resident and preceptor composite scores for the 12 quality criteria used to assess the residency assessment program. Eleven of the 12 quality criterion demonstrated acceptable Cronbach’s alpha (α > 0.60), indicating an analysis of the composite quality criteria from each of the four indicator questions to be appropriate [10]. Cronbach’s alpha for Costs and Efficiency (α = 0.35) suggests that the four items used to measure this construct lacked internal consistency. Baartman and colleagues regarded criteria receiving scores at 65 or higher to be of good quality, scores between 30 and 64 to be of medium quality, and scores below 30 to be poor [9]. Median composite scores for the 12 quality criteria ranged from 43.00 to 84.75, with Comparability (84.75, 25.00–100.00) and Transparency (82.50, 25.50–100.00) rated the highest. Educational Consequences (43.00, 10.75–96.50) and Costs and Efficiency (64.25, 37.00–95.25) were the only two composite quality criteria that fell below a score of 65. While median responses tended to exceed 65, the ranges for composite scores and individual items showed wide variability in responses.

**Table 3:** Resident and preceptor evaluation of the residency assessment program based on the 12 quality criteria of the CAP. Two example items for each four-item criterion (taken from the resident survey) are provided.

Criterion ^a^	N	Mean, Median (Range)	α
Acceptability	51	69.28, 69.25 (8.25–100)	0.90
The criteria I was evaluated on as a part of the residency assessment program were appropriate for my training	51	75.25, 76.00 (13.00–100)	
All methods used to evaluate me during residency were appropriate	51	65.86, 68.00 (0.00–100)	
Authenticity	51	74.92, 80.13 (41.75–100)	0.87
The working conditions in which I was assessed during residency resemble the conditions of the workplace	51	72.49, 80.00 (0.00–100)	
The competencies on which I was assessed during my residency program are those I need to be successful in the workplace	51	72.29, 76.00 (0.00–100)	
Cognitive Complexity	51	73.09, 73.25 (31.25–100)	0.91
The assessment program placed an emphasis on the thought processes involved in my process for providing patient care	51	70.65, 73.00 (25.00–100)	
My reasoning and thought process was assessed within the assessment program	51	75.24, 75.00 (37.00–100)	
Comparability	49	78.62, 84.75 (25.00–100)	0.92
The activities I was evaluated on were the same from month to month for a similar type of rotation	49	79.41, 75.00 (37.00–100)	
The process by which I was evaluated for each criterion was the same from month to month for a similar type of rotation	49	81.67, 87.00 (25.00–100)	
Costs and Efficiency	51	64.27, 64.25 (37.00–95.25)	0.35
I believe the time required for each assessment method utilized during my residency was appropriate.	51	56.59, 53.00 (11.00–100)	
I would have been willing to put in more time towards the assessment and feedback aspects of residency if I perceived them to have a greater value.	51	73.84, 79.00 (4.00–100)	
Educational Consequences	51	45.26, 43.00 (10.75–96.50)	0.66
The assessment process during residency motivated me to learn more during residency	51	44.29, 45.00 (0.00–97.00)	
My assessment during residency impacted the objectives of subsequent learning experiences during my residency	51	43.68, 50.00 (0.00–100)	
Fairness	51	76.59, 78.88 (36.50–100)	0.71
The residency assessment program during my residency was fair	51	75.98, 77.00 (0.00–99.00)	
I was evaluated with various assessment methods (*i.e*., verbal feedback, midpoint evaluations, portfolio)	51	81.00, 87.00 (31.00–100)	
Fitness for Purpose	51	72.68, 75.50 (25.50–100)	0.75
The activities I was assessed on matched stated educational goals	51	76.82, 76.00 (31.00–100)	
The assessment program covered assessment of all competency areas required in my residency training	51	69.67, 75.00 (0.00–100)	
Fitness for Self-Assessment	51	69.43, 72.75 (10.50–100)	0.77
The assessment methods during my residency stimulated me to self- assess my learning	51	67.19, 70.00 (11.00–100)	
I received feedback that fostered my personal development	51	66.35, 70.00 (0.00–100)	
Meaningfulness	49	72.39, 75.13 (3.75–100)	0.85
The assessment methods used during my residency enhanced my learning	49	65.35, 70.00 (4.00–100)	
The competencies I was assessed on are meaningful to me	49	69.94, 76.00 (5.00–100)	
Reproducibility of Decisions	46	76.02, 77.88 (37.50–100)	0.80
My evaluations were consistent throughout the year	46	67.20, 72.50 (3.00–100)	
I am assessed the same way in several different work situations	46	83.72, 82.00 (12.00–100)	
Transparency	46	77.19, 82.50 (25.50–100)	0.93
I was aware of the assessment criteria used during my residency	46	79.24, 88.50 (22.00–100)	
I was aware of the assessment methods used during my residency	46	76.35, 89.50 (0.00–100)	

^a^ All items measured on a continuous scale ranging from 0 (*Not at all)* to 100 (*Completely*); Each criterion consists of four survey items.

Mann-Whitney U tests were used to examine responses between residents and preceptors and between individuals who completed residency training only at the institution and those that completed residency training elsewhere (Table 4). When compared to those who completed residency training only at the academic medical center, those who completed residency training elsewhere scored the assessment program higher on Meaningfulness (83.13, 32.75–100.00 *versus* 70.88, 3.75–90.00, *p* = 0.01) and Comparability (90.00, 55.00–100.00 *versus* 77.38, 25.00–100.00, *p* = 0.04). No significant differences were found based on number of years of precepting, year of residency completion, or experience precepting elsewhere.

### 3.3. Discussion

Competency development in the workplace is a priority for the pharmacy profession, with emphasis on identifying best practices for assessing the achievement of professional competencies. In this study, the perceived quality of the assessment program for pharmacy residents at an academic medical center in the United States was studied as an important step towards improving assessment strategies and understanding how assessment practices align with desired outcomes. The results of the 48-item, 12 quality criteria survey demonstrated the strengths of the assessment program, identified opportunities to focus improvement efforts on specific aspects of the residency assessment program, and provides a framework for replicating assessment program evaluations in other workplace settings.

Based on resident and preceptor scores, it appears that the assessment program could benefit from strategies that improve the Educational Consequences of the assessments. Low scores for this quality indicator suggest that the current assessments provide little motivation for residents to learn more, may hinder achievement of desired learning outcomes, and offer limited incentive for learners to incorporate feedback from faculty. More broadly, Educational Consequences concerns the impact of the competency assessment program on learning and instruction, including how the individuals view the goals of education and adjust their learning activities accordingly [8]. In the absence of educational consequences, it is difficult for assessments to exert much influence on learning. Given the integrated nature of assessment and learning, priority should be placed on improving the quality of education consequences and improving the effects of the assessment program on participants.

The quality criteria Costs and Efficiency scored below 65, varied widely at the item level, and lacked internal consistency. As a construct, Costs and Efficiency broadly relates to the time and resources needed to carry out the assessment program compared to the benefits, meaning that any additional time spent on the assessment program should be justified by the positive effects of assessment, including improvements in learning, teaching, and motivation [5]. The results of this study suggest that, although the current residency assessment process is perceived as time-intensive, participants may be willing to invest more time for better value. In making improvements to workplace learning, re-evaluating how time is spent on the assessment program could be advantageous with specific consideration to the benefit that the assessment provides to the learner.

Remaining quality criteria were generally rated highly by the study participants. Comparability and Transparency were ranked the highest by residents and preceptors, indicating a consistency in working conditions and criteria evaluated and understanding by all parties (e.g., learners, preceptors, program directors, mentors) involved. In general, these quality criteria are strengths of the pharmacy residency assessment program and should be embraced while considering programmatic changes. At the same time, however, large ranges in responses at the criteria and item level suggest that not all of these criteria may be equally agreed upon. This variability indicates differing perceptions of what constitutes assessment quality at the individual level, suggesting that those overseeing workplace learning should consider approaches to ensuring that individuals are getting what they need from assessments to achieve desired competencies.

Group comparisons in this study provide additional insight into the quality of the assessment program. First, no significant differences were found in perceived quality of the assessment program between residents and preceptors, suggesting that residents and preceptors view the quality of the assessment program similarly. However, significant differences were found between individuals who had residency training outside of the institution versus those who did not. Those who completed residency elsewhere perceived the assessment program as more meaningful and comparable than those who completed residency training at the academic medical center. Meaningfulness relates to the value that all stakeholders place in the assessment program (e.g., learners, leaders, employers) and these findings suggest that those that trained elsewhere found the academic medical center’s assessment program to be more meaningful and valuable to learners. Comparability concerns the extent to which a CAP is conducted consistently and responsibly and these findings suggest that those with training elsewhere perceived the assessment program at the academic medical center as more consistent and responsible. Soliciting the experience of residents and preceptors with experience at other institutions may help uncover the source of these differences.

**Table 4:** Group comparisons for scores on the 12 quality criteria of the CAP, by position (resident, preceptor) and training location (at the institution, elsewhere).

Criterion	Residents (*n* = 23)	Preceptors (*n* = 28)		Residency Only at Institution (*n* = 30)	Residency Training Elsewhere (*n* = 21)	
	Median (Range)	Median (Range)	*p*-value	Median (Range)	Median (Range)	*p*-value ^a^
Acceptability	76.50 (8.75–100)	67.50 (52.50–95.00)	0.08	67.50 (8.75–95.00)	75.00 (52.50–100.00)	0.12
Authenticity	81.75 (43.75–100)	78.25 (41.75–95.25)	0.72	77.50 (43.75–95.25)	83.75 (41.75–100.00)	0.09
Cognitive Complexity	77.25 (50.75–100)	71.75 (31.25–94.50)	0.17	74.38 (31.25–94.50)	72.25 (50.00–100.00)	0.32
Comparability	76.12 (25.00–100)	88.75 (59.25–100)	0.09	77.38 (25.00–100.00)	90.00 (55.00–100.00)	0.04
Costs and Efficiency	59.62 (37–95.25)	68.12 (38.25–85.00)	0.21	62.50 (39.75–78.50)	76.25 (37.00–95.25)	0.16
Educational Consequences	43.75 (10.75–96.50)	42.25 (12.25–79.25)	0.91	43.75 (12.25–83.75)	42.38 (10.75–96.50)	0.83
Fairness	75.75 (36.50–100)	80.75 (43.00–100.00)	0.41	78.75 (36.50–100.00)	83.00 (43.00–100.00)	0.87
Fitness for Purpose	80.87 (25.50–100)	74.87 (45.00–92.75)	0.42	75.00 (25.50–92.75)	76.25 (45.00–100.00)	0.53
Fitness for Self- Assessment	69.75 (10.50–100)	74.00 (26.25–93.75)	0.89	68.75 (10.50–93.75)	75.75 (37.25–100.00)	0.13
Meaningfulness	75.00 (3.75–100)	75.25 (32.75–91.00)	0.91	70.88 (3.75–90.00)	83.13 (32.75–100.00)	0.01
Reproducibility of Decisions	80.00 (37.50–100)	76.00 (62.50–99.75)	0.96	75.25 (37.50–99.75)	80.38 (41.75–100.00)	0.42
Transparency	86.50 (34.00–100)	78.00 (25.50–98.75)	0.08	78.00 (25.50–100.00)	87.78 (51.25–100.00)	0.15

^a^All group comparisons examined using Mann-Whitney U. Significance set at *p* < 0.05.

This study had several limitations. Due to the uniqueness of assessment programs and variability associated with each institution, the study was limited to a single institution. While this may limit generalizability to other institutions, this study provides a framework and process for evaluating the quality of the assessment program that other institutions could implement. The length of the survey tool itself appears to be a limitation as there were 7 incomplete responses excluded from the study. A more accurate representation of the time required for the survey or designated work time to complete the survey may have improved response rates and increased the sample size. The small sample size may have limited the ability to detect differences in subsets of this sample due to a lack of power and *p* values ≤ 0.20 may warrant additional study. Despite these limitations, it is believed that the results from this study provide critical insight into the CAP and will be useful for informing programmatic improvement.

This work is a first step in fully understanding the quality and impact of the assessment program for trainees at an academic medical center in the United States. Future work should extend this study to evaluate how assessment feedback is incorporated into practice (e.g., patient care) or intermediary outcomes. In addition, this research evaluated the residency assessment program at the program level and provided limited information about individual assessment methods or instruments, leaving additional work to be done toward identify gaps and weaknesses associated with single assessment approaches or instruments. Future research should also extend this framework and process to additional institutions in an effort to identify trends and differences in assessment program quality that could inform the profession moving forward.

## 4. Conclusions

Interest in processes that support demonstration of achieved competencies in the pharmacy workplace is growing. Overall, the assessment program used to evaluate workplace learning in this study was perceived to be effective by pharmacy residents and preceptors. Results suggest that the focus of improvement in the residency assessment program should target Educational Consequences, ensuring that assessments promote motivation and incentives. Further, workplace learning programs should consider differences associated with training outside of the institution, as those with experiences in other workplace environments are likely to perceive assessments differently than those with training only at the institution.
